# Shining the Path of Precision Diagnostic: Advancements in Photonic Sensors for Liquid Biopsy

**DOI:** 10.3390/bios15080473

**Published:** 2025-07-22

**Authors:** Paola Colapietro, Giuseppe Brunetti, Carlotta Panciera, Aurora Elicio, Caterina Ciminelli

**Affiliations:** Optoelectronics Laboratory, Politecnico di Bari, Via E. Orabona 6, 70125 Bari, Italy; p.colapietro@phd.poliba.it (P.C.); giuseppe.brunetti@poliba.it (G.B.); c.panciera1@phd.poliba.it (C.P.); a.elicio1@phd.poliba.it (A.E.)

**Keywords:** liquid biopsy, label-free photonic biosensors, circulating tumor biomarkers, cancer detection

## Abstract

Liquid biopsy (LB) has gained attention as a valuable approach for cancer diagnostics, providing a minimally invasive option compared to conventional tissue biopsies and helping to overcome issues related to patient discomfort and procedural invasiveness. Recent advances in biosensor technologies, particularly photonic sensors, have improved the accuracy, speed, and real-time capabilities for detecting circulating biomarkers in biological fluids. Incorporating these tools into clinical practice facilitates more informed therapeutic choices and contributes to tailoring treatments to individual patient profiles. This review highlights the clinical potential of LB, examines technological limitations, and outlines future research directions. Departing from traditional biosensor focused reviews, it adopts a reverse-mapping approach grounded in clinically relevant tumor biomarkers. Specifically, biomarkers associated with prevalent cancers, such as breast, prostate, and lung cancers, serve as the starting point for identifying the most suitable photonic sensing platforms. The analysis underscores the need to align sensor design with the physicochemical properties of each biomarker and the operational requirements of the application. No photonic platform is universally optimal; rather, each exhibits specific strengths depending on performance metrics such as sensitivity, limit of detection, and easy system integration. Within this framework, the review provides a comprehensive assessment of emerging photonic biosensors and outlines key priorities to support their effective clinical translation in cancer diagnostics.

## 1. Introduction

Cancer remains a major cause of mortality worldwide, with World Health Organization (WHO) statistics reporting 8.8 million deaths globally in 2015 [1] and 10.3 million in 2020 [2]. Driven by demographic shifts, including population aging and growth, the global cancer burden is expected to increase significantly. Annual cases are projected to reach 19 million by 2050 [3], with trends varying across countries and regions. Cancer develops through the gradual accumulation of genetic mutations, frequently involving key regulatory genes associated with cellular growth, proliferation, and apoptosis, thereby initiating carcinogenesis [4]. Detecting tumor-specific genetic mutations via molecular analysis plays a key role in diagnosis and prognosis, as well as guiding treatment decisions.

Despite ongoing technological advances, standard clinical protocols for tumor diagnosis still rely primarily on tissue biopsy [5,6]. This procedure is typically performed after detecting potentially malignant abnormalities through imaging modalities like ultrasound, CT scans, or MRI. Biopsy methods, largely unchanged since their origin in medicine [7], involve the physical removal of tissue through surgical, core needle, or fine needle techniques [8]. Tumor tissue remains the primary source for diagnostic, prognostic, and therapeutic information. It allows clinicians to distinguish benign from malignant conditions and assess biomarkers of clinical relevance [9,10]. However, tissue biopsies are invasive and often unfeasible for tumors that are anatomically inaccessible or infiltrative [11]. They offer only a limited, time-specific view of tumor status, which may not reflect its full genomic complexity, especially given the dynamic and heterogeneous nature of cancer. Repeated samples may be needed, which can reduce patient compliance.

To overcome limitations such as sampling bias, procedural invasiveness, and poor suitability for longitudinal monitoring, liquid biopsy (LB) has emerged as a complementary and less invasive alternative. It enables the collection of tumor-derived information and supports real-time monitoring of cancer evolution. First introduced by K. Pantel et al. [12] in 2010, this technique enables the collection and analysis of biological fluids, primarily blood, to extract clinically relevant information for diagnostic, prognostic, and monitoring purposes. This approach is based on the analysis of the tumor circulome [13], which encompasses a heterogeneous set of tumor-derived components circulating in bodily fluids. These include circulating tumor proteins, cell-free tumor nucleic acids, such as cfDNA and cfRNA [13], circulating tumor cells (CTCs) [14], cell-free microRNAs (cfmiRNAs) [15], mRNA [16], long noncoding RNA [16], small extracellular vesicles (EVs) [14], and tumor-educated platelets (TEPs) [17]. Collectively, these elements represent a rich and dynamic source of biomarkers, offering a more comprehensive and real-time view of tumor-based biology than conventional tissue sampling. A detailed overview of these circulating biomarker classes and their diagnostic relevance is provided in Section 2.

The LB workflow can be tailored to the specific physiochemical properties of each biomarker class. This flexibility enables the early detection of metastatic disease and supports personalized treatment strategies. Compared to tissue biopsy, LB is less invasive, enables repeated testing, ensures greater patient comfort, and yields faster results. Moreover, it is applicable regardless of tumor location and enables dynamic monitoring of therapeutic efficacy and resistance mechanisms [18]. A comparative overview of the main advantages and limitations of both approaches is presented in Table 1.

While LB enables dynamic and non-invasive molecular profiling, it lacks the morphological and architectural details provided by histological examination. For this reason, LB should be viewed as a complementary approach rather than a replacement for tissue-based analysis. Comparative studies often report discrepancies between the two methods: for instance, only 10 out of 45 mutations overlapped in one multi-cancer study [19].

Overall, tissue biopsy remains the preferred option when tumors are accessible and morphological characterization is required. In contrast, LB is particularly suitable for early detection, mutational profiling in metastatic cancer, and real-time monitoring of treatment-induced molecular changes. Ideally, combining both approaches enables robust, longitudinal assessment of tumor evolution and improves cancer management.

Given LB’s clinical potential, there is a growing need for advanced analytical platforms capable of reliably detecting and quantifying circulating biomarkers [20]. To fully exploit its advantages of LB, technologies with rapid, sensitive, and cost-effective detection capabilities are required. Biosensor-based technologies have increasingly been explored to meet these analytical demands. Among them, photonic biosensors are especially promising due to their intrinsic optical properties, which enable label-free, highly sensitive detection without the need for reagents. Moreover, their compatibility with on-chip integration makes them well suited for decentralized diagnostic applications, including Point-Of-Care (PoC) settings [21,22]. These platforms can identify multiple biomarkers simultaneously, directly from minimally processed samples, making them valuable tools for real-time clinical evaluation and localized cancer diagnostics [23,24]. The review is structured to provide a clinically grounded and technologically informed perspective on the role of photonic biosensors in liquid biopsy. First, we outline the diagnostic potential and current limitations of LB, emphasizing its comparative advantages over conventional tissue biopsy. Next, we present an overview of the most prevalent cancer types and their associated circulating biomarkers. Finally, we examine the major classes of existing photonic biosensors, providing a biomarker-specific comparative assessment that highlights their relative strengths, limitations, and suitability for clinical implementation. This structure reflects the multidisciplinary nature of the review, which not only examines photonic technologies in depth but also integrates a biologically grounded framework, resulting in a comprehensive and heterogeneous analysis that bridges sensor design with clinical relevance. By aligning clinical needs with device-level functionalities, this work aims to identify the most promising technologies and highlight key challenges for future clinical translation.

## 2. Tumor Biomarkers as Indicators in Cancer Diagnosis

Various tumor-derived biomarkers can be accessed through LB strategies, including circulating tumor cells (CTCs) [14], extracellular vesicles (EVs) [14], circulating tumor DNA (ctDNA) [13], and microRNAs (miRNAs) [16]. These indicators offer a non-invasive route for cancer detection and follow-up. However, accurately identifying these targets remains technically challenging due to their low abundance and the complex composition of biological fluids. To overcome these limitations, photonic biosensors have gained attention as effective analytical platforms, thanks to their exceptional sensitivity and specificity at the molecular level. Moreover, photonic sensing platforms can overcome key limitations of conventional methods by improving detection limits and reducing sample preparation requirements. To contextualize the analytical challenges and clinical relevance of LB biomarkers, Table 2 presents a structured overview of five major biomarker classes (CTCs, EVs, exosomes, ctDNA, and miRNAs), along with their associated cancer types. For each cancer–biomarker pair, the table reports the typical concentration ranges in relevant biofluids (e.g., blood, plasma, and urine) while accounting for matrix-dependent variability. It also reports conventional diagnostic technologies and their respective limits of detection. 

**Table 2:** Summary of key liquid biopsy biomarkers across cancer types: associated biological fluids, typical concentration, conventional diagnostic methods, and limits of detection.

Biomarker	Associated Cancer Type	Biological Fluid	Typical Concentration	Diagnostic Technology	LoD	Refs.
CTC	Metastatic lung, prostate, pancreatic, and colon cancers	Peripheral whole blood	1–5 cells per 7.5 mL	CTC-chip	5–1281 CTCs/mL	[25]
	Breast cancer	Peripheral whole blood	500 CTCs per 7.5 mL	NP-HBCTC-Chip	6–12 CTCs/mL	[26,27]
	Prostate, ovarian, breast, gastric, colorectal, bladder, and renal cancers	Peripheral whole blood	1–5 cells per 7.5 mL	CellSearch system	60 ± 693 CTCs per 7.5 mL	[28]
EVs	Prostate cancer	Urine	~10^10^ EVs per mL	Ultracentrifugation	4.13 ± 3 × 10^11^ particles/mL	[29,30]
	Breast cancer	Plasma	~10^10^ EVs per mL	DNA aptamer-based system	0.21–1.87 × 10^9^ particles/mL	[30,31]
Exosome	Melanoma	Plasma	~10^10^ EVs per mL	^new^ExoChip	2.79 × 10^8^ particles/mL	[30,31]
	Lung cancer	Plasma	~10^10^ EVs per mL	^new^ExoChip	2.89 × 10^8^ particles/mL	[30,32]
ctDNA	Breast, prostate, NSCLC, ovarian, and gastric cancers, lymphoma, and glioblastoma	Plasma	0.01–0.1% allele frequency	BEAMin TAm-Seq CAPP-Seq ddPCR	0.01% allele frequency (ddPCR)	[33]
miRNA	Breast, lung, and colorectal cancers	Serum, plasma	~pg–ng/mL	Immunomagnetic Exosomal RNA (iMER) technology	~pg–ng/mL	[34]

This comparison underscores the heterogeneity in biomarker abundance and the limitations of existing detection methods. It further highlights the rationale for integrating photonic biosensors into next-generation diagnostic workflows.

### 2.1. CTCs

CTCs originate from either primary or secondary tumor sites and play a pivotal role in metastatic progression [35]. However, their extreme rarity (1 CTC per 10^6^–10^7^ leukocytes) poses significant detection challenges [36]. Notably, multiple studies have reported that their abundance typically ranges between one and five cells in a 7.5 mL blood sample [35]. Among the available technologies, CellSearch relies on magnetic beads functionalized with specific antibodies for CTC enrichment [37]. In contrast, label-free photonic biosensors, which exploit dielectric properties, particle size, or refractive index variations, represent promising alternatives with improved sensitivity and real-time analytical capabilities.

### 2.2. Extracellular Vesicles

Extracellular vesicles (EVs) constitute a diverse population of lipid bilayer enclosed particles secreted by multiple cell types, including cancer cells, and are readily found in various bodily fluids. The composition of these vesicles, encompassing proteins, lipids, and nucleic acids, reflects the molecular characteristics associated with the physiological or pathological condition of their cells of origin. For this reason, EVs represent ideal biomarkers: the structure and functions of a tumor cell differ significantly from those of a healthy cell, and these differences are clearly reflected in the composition and cargo of the respective EVs they release. In addition to their diagnostic potential, EVs play an active role in cancer biology, contributing to tumor progression, drug resistance, and intercellular communication. Among EV subtypes, exosomes (30 to 150 nm) are particularly relevant due to their molecular content and role in cancer progression [38]. Traditional isolation methods such as ultracentrifugation or immunoaffinity capture are labor intensive and lack specificity [39]. As a result, research is actively focused on developing more efficient isolation techniques that also enable the selective isolation of tumor-derived vesicles from those originating from other cells, such as photonic biosensors.

### 2.3. Circulating Tumor DNA (ctDNA)

Circulating tumor DNA (ctDNA) constitutes a minor portion of the total cell-free DNA (cfDNA) and reflects the mutational profile characteristic of the tumor. Its levels vary by cancer type and stage, typically remaining below 0.01% of total cfDNA [40]. Standard detection relies on digital polymerase chain reaction (dPCR), bead emulsion amplification magnetics (BEAMing) technology, tagged-amplicon deep sequencing (TAmSeq) [41], and nontargeted next-generation sequencing (NGS) [42], which have limited sensitivities at low concentrations. Photonic biosensors, leveraging nanoplasmonics or photonic crystals, can improve detection limits and provide rapid, amplification-free identification of clinically relevant mutations.

### 2.4. Circulating miRNA

miRNAs are short, non-coding (14–20 nucleotides) molecules involved in gene regulation and are stable in biological fluids, making them ideal LB biomarkers [43]. However, commonly employed methods for detecting miRNAs, including quantitative real-time polymerase chain reaction (qRT-PCR), Northern blotting, and oligonucleotide microarray, suffer from limitations in cost, processing time, and sensitivity, which restrict their applicability in in vitro diagnostics (IVD) [43,44]. With high specificity and sensitivity, photonic biosensors (e.g., microcavity resonators and silicon photonic waveguides) can detect miRNAs without the need for fluorescent or enzymatic labeling.

### 2.5. Main Approaches for Detecting Cancer Biomarkers: From Gold Standards to Liquid Biopsy

Building on the previous discussion, this section compares conventional diagnostic methods with emerging LB technologies to address key challenges in precision oncology.

Advancements in precision medicine are reshaping the diagnostic landscape, fostering a shift toward patient-specific strategies guided by molecular profiling [45,46]. Alongside novel therapeutic approaches, such as cell engineering and nanotechnologies, there is an increasing demand for diagnostic systems that are rapid, sensitive, cost effective, and suitable for early detection and longitudinal monitoring [47]. Although conventional techniques such as histopathology, immunohistochemistry (IHC), ELISA, and PCR remain essential, their limited scalability, long turnaround times, and reliance on centralized laboratories hinder their broader application in personalized oncology. Although techniques like PCR and immunoassays offer high sensitivity and specificity, their reliance on complex infrastructure and slow turnaround times limits their use in high-throughput and real-time applications.

Before 2010, commonly adopted techniques for the extraction of circulating biomarkers, particularly EVs and exosomes, included ultracentrifugation, magnetic bead separation based on immunoaffinity, and various commercial kits [47,48]. Both classical and density gradient-based approaches provide moderate purity, with higher levels achieved using gradient-based separation. This method ensures a sufficiently high yield of exosomes (~1.5 × 10^9^ particles/mL of concentrated conditioned medium) and RNA (~0.2 µg per 10^8^ particles), while the protein yield is moderate (~0.5 µg per 10^8^ particles). To enhance purity, size exclusion techniques using High-Performance Liquid Chromatography (HPLC) are often employed. However, these require specialized equipment and are relatively expensive. Ultracentrifugation remains more affordable per sample compared to chemical precipitation methods and has a shorter processing time (~4 h versus up to 20 h) [47]. Nevertheless, to address these drawbacks, immunoaffinity and commercial kits were introduced and are now increasingly adopted. The immunoaffinity method shows ~75% efficiency for exosomes and ~90% for miRNAs [49]. Commercial kits (e.g., ExoQuick or Exo-spin) isolate exosomes in ~1 h and offer high yield, although their purity varies. Some formulations achieve yields up to 150× greater than ultracentrifugation, while others balance yield (~70%) and purity (~95%), surpassing conventional methods by up to 10× [50,51,52]. Despite their advantages, these methods still face challenges such as cost, multistep protocols, and reliance on specialized infrastructure.

These constraints have fueled the rise of LB as a promising alternative for minimally invasive detection and monitoring of tumor-specific biomarkers. Capture often exploits the physical or chemical properties of the target to achieve high sensitivity, low contamination, and structural preservation. For example, the CellSearch system employs immunomagnetic beads and achieves capture efficiencies ranging from 60% to 90%; however, it remains limited by extended processing times and significant contamination with leukocytes [53]. High-throughput, low-contamination isolation strategies have also been demonstrated, such as the microfluidic device developed by T.W. Lo et al. [54], which processes up to 10 mL/h of biological sample while preserving the structural and functional integrity of extracellular vesicles from downstream analysis. Following the capture phase, tumor biomarkers are detected and quantified using techniques including fluorescence labeling or optical, acoustic, or electrochemical sensing, achieving detection sensitivities down to the femtomolar (fM) range for specific nucleic acids and proteins [55]. The final phase involves comprehensive analysis of the isolated biomarkers, ranging from enumeration and molecular characterization to bioinformatic profiling, enabling their use in predictive screening, early diagnosis, disease monitoring, and therapeutic stratification in accordance with defined clinical protocols.

To enhance clinical integration, researchers are optimizing microfluidic platforms, lab-on-chip (LoC), and micro total analysis systems (µTASs), which combine capture, detection, and analysis within a compact chip handling picoliter volumes [56,57]. Until now, the CellSearch system is the first and only Food and Drug Administration (FDA)-approved LoC for CTC enrichment and enumeration in whole blood [58]. Requiring just 7.6 mL of blood, it enables detection of a single CTC with >99% specificity and sensitivity ranging from 50 to 70%, depending on the cancer type. Despite slightly lower sensitivity than some alternatives (~76%) [59,60], it offers robust automation, phenotypic and molecular characterization, and clinical reliability, with a critical threshold set at 3–5 CTCs per sample [37].

Despite LB’s potential, biological challenges remain low biomarker abundance (e.g., ctDNA < 0.01% in early disease, CTCs < 5 per 7.5 mL), heterogeneity (e.g., epithelial–mesenchymal transition), and dependence on the biological matrix, with blood still being the most used [27,54,61].However, the performance of current LoC systems is often limited by insufficient biomarker recovery, contamination, and a lack of analytical precision. In this context, biosensor technologies emerge as a powerful solution, offering miniaturization, integration, low sample consumption, and enhanced sensitivity. Section 4 provides an overview of biosensing platforms, particularly photonics-based technologies, as core enablers of next-generation diagnostic devices, outlining their key components and operating principles.

### 2.6. Liquid Biopsy Sampling Techniques and Their Integration with Photonic Biosensors

The accuracy and reliability of LB heavily depend on the efficiency of sampling techniques used to isolate circulating biomarkers from body fluids. Various strategies have been developed to address preanalytical challenges, including invasiveness, sample degradation, and logistical complexity [14,18]. Among these, venous blood draw remains the clinical standard; however, it is limited by the need for trained personnel and centralized processing. To overcome these barriers, alternative sampling approaches have been developed to improve the clinical applicability and decentralization of LB.

Capillary blood microsampling, for example, enables minimally invasive, low-volume sampling suitable for self-collection and remote settings. High sample integrity, analytical compatibility, and simplified storage and transport have been demonstrated by techniques such as dried blood spot (DBS), volumetric absorptive microsampling (VAMS), and commercially available devices like HemaPEN^®^ (Traian Scientific and Medical, Melbourne, Australia) and Capitainer^®^ (Capitainer Ab, Solna, Sweden).These technologies have been extensively reviewed by Thangavelu et al. [62], who underscore their potential to enable frequent and patient-centric monitoring of biomarkers while reducing biohazard risk and preanalytical variability. As an additional advancement, Septa et al. [63] introduced the BioSampler device, designed to preserve and transport viral specimens in a dried format while maintaining compatibility with nucleic acid amplification techniques (NAATs). Originally intended for molecular testing of SARS-CoV-2, the BioSampler showed robust RNA preservation for up to 28 days under elevated temperature conditions, performing comparably to standard transport media in qPCR assays. Its use illustrates how dried-format sampling technologies can be adapted for broader molecular diagnostic applications, including liquid biopsy in oncology [63]. Beyond blood-based approaches, emerging sampling techniques include saliva, urine, cerebrospinal fluid (CSF), and pleural effusion collection, which provide access to complementary biomarker profiles with variable degrees of invasiveness and clinical utility. The integration of these strategies with photonic biosensors is a critical step toward developing decentralized PoC platforms. By ensuring sample compatibility, stability, and minimal preparation, these techniques facilitate the direct analysis of low-abundance biomarkers using label-free optical detection.

## 3. Prevalent Cancer Types and Associated Biomarkers

Cancer is a highly heterogeneous disease, comprising over 277 distinct malignancies with diverse histopathological and molecular features [64]. This heterogeneity poses considerable challenges for early diagnosis and effective therapeutic interventions. Among the many cancer types, breast, prostate, and lung cancers consistently rank as the most frequently diagnosed worldwide and are associated with a high clinical burden. The incidence and mortality statistics provided by CANCER TODAY [65] are the results of collaborative data collection involving the National Cancer Institute (NCI), and the North American Association of Central Cancer Regis-tries (NAACCR) [66,67]. The high incidence of these tumor types, coupled with the availability of well-characterized circulating biomarkers such as HER2, PSA, and CEA, makes them exemplary reference models for validating liquid biopsy platforms that incorporate photonic biosensing technologies.

While this section primarily focuses on the key biomarkers relevant to these cancer types, emerging evidence also highlights the growing relevance of LB in other malignancies with distinct diagnostic challenges. Notably, brain and cervical cancers, though markedly different in terms of histology and clinical course, share specific barriers that underscore the need for minimally invasive diagnostic alternatives. In both cases, direct access to tumor tissue is often limited due to anatomical constraints or procedural risks, making repeat tissue biopsies clinically impractical. Consequently, novel sampling strategies, such as LB and alternative biological fluids, are being explored. In brain cancers, particularly gliomas, the analysis of cfDNA and tumor-specific mutations in cerebrospinal fluid outperforms plasma-based approaches. Similarly, in cervical cancer, especially in HPV-driven lesions, recent studies have demonstrated the feasibility of detecting circulating HPV-DNA and microRNA [68,69]. In pancreatic cancer, where early diagnosis is often hindered by the anatomical inaccessibility of the tumor and its aggressive course, ctDNA and exosomal RNA have been investigated as potential biomarkers for early detection and treatment response assessment [70]. Although still under validation, these developments demonstrate the expanding applicability of LB across a broader range of cancer types, reinforcing its role as a versatile tool for personalized oncology.

### 3.1. Breast Cancer

Among women, breast cancer (BC) represents the most frequently diagnosed type of malignancy, contributing to roughly 11.7% of all female cancer cases worldwide. Although rare, it may also occur in men [71]. From a clinical perspective, breast cancer subtyping relies on immunohistochemical (IHC) analysis of critical biomarkers, including estrogen and progesterone receptors (ER and PR), the proliferation index Ki-67, and human epidermal growth factor receptor 2 (HER2) [72]. To complement imaging and tissue biopsy, LB allows for the detection of key circulating biomarkers, including HER2, cancer antigen 15-3 (CA 15-3), carcinoembryonic antigen (CEA), and plasminogen activator-1 (PAI-1). Conventional LB methods include colorimetric and fluorescence assays, electrochemical sensors, chromatographic techniques, and ELISA [73]. Notably, CEA identification in nipple aspirate fluid (NAF) demonstrated higher specificity than serum-based analysis, underscoring the potential of localized biosensing strategies.

### 3.2. Prostate Cancer

Prostate cancer (PC) is among the most prevalent tumors and a leading cause of cancer-related mortality in men, with approximately 375,000 deaths reported globally each year. Its incidence increases with age, and diagnosis often occurs at advanced stages due to its asymptomatic progression. Traditional diagnostic tools such as Digital Rectal Examination (DRE) and Positron Emission Tomography (PET) imaging are limited by sensitivity, invasiveness, and cost. To overcome these drawbacks, serum and urine have become the preferred matrices for non-invasive testing, reducing discomfort and procedural risks [74]. Among the most studied biomarkers, prostate-specific antigen (PSA) remains the clinical reference standard, even though its specificity is reduced by benign prostatic conditions [74].

### 3.3. Lung Cancer

Lung cancer ranks among the most aggressive tumor types from a clinical standpoint, primarily because early, asymptomatic growth delays diagnosis until advanced stages [75]. Both non-small-cell lung cancer (NSCLC) and small-cell lung cancer (SCLC) are commonly associated with metastatic spread at the time of diagnosis, which severely compromises prognosis and therapeutic efficacy. Conventional diagnostic methods, including tissue biopsy, CT imaging, and sputum cytology, offer varying degrees of sensitivity and specificity but are often invasive, costly, and unsuitable for longitudinal monitoring [43]. LB has gained recognition as a less invasive approach capable of identifying lung cancer-associated biomarkers in biological fluids such as blood, sputum, bronchial aspirates (BASs), bronchoalveolar lavage (BAL), and pleural effusions. Within the diagnostic framework of lung cancer, molecular biomarkers such as KRAS mutations and HER2 amplification are among the most extensively investigated targets for diagnostic and prognostic purposes. Their detection with emerging photonic biosensors could advance diagnosis and guide personalized therapy.

## 4. Photonic Biosensor Technologies for Liquid Biopsy Applications

**Figure 1:**
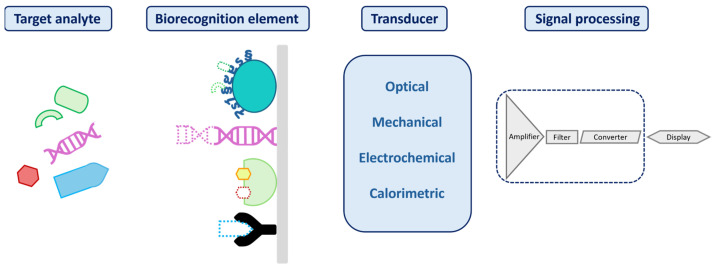
Illustration of a biosensor structure, with its three key components: a biorecognition element; a transducer to convert the biorecognition interaction into a detectable signal; and a processor responsible for amplifying, processing, and presenting the signal.

A biosensor is a compact analytical device designed to translate biochemical or chemical interaction into a measurable output signal, commonly optical, electrical, or acoustic, that correlates with the concentration of a specific target. The output originates from a selective recognition event, such as a molecular binding or reaction involving the analyte of interest [76]. Figure 1 illustrates a typical biosensor, highlighting its three essential components and their functional roles in the sensing process.

Biosensors can be classified by their biorecognition element (e.g., enzymes, antibodies, or aptamers) or by their transduction method (electrochemical [77], optical [78], electronic [79], acoustic [80], or mechanical [81]). A summary of electrochemical, optical, and mechanical biosensing strategies for LoC application is provided in Table 3.

Among these, photonic biosensors stand out for their label-free, high sensitivity (S), and multiplexing capabilities, features well suited to liquid biopsy workflows. This class of biosensors convert micro- and nanoscale changes in optical properties, such as phase, amplitude, polarization, or frequency, into quantifiable signals.

**Table 3:** Overview of biosensor types for liquid biopsy: key characteristics, benefits, and limitations.

Transducer Type	Key Characteristics	Strengths	Limitations	Performance
Electrochemical	Enables real-time monitoring, typically within several hundred seconds Common in PoC systems. Short operational lifespan Cost-effective microelectrode production	Straightforward operation Suitable for continuous analysis across multiple targets Economic	Devices tend to be bulky Susceptible to environmental fluctuations (e.g., temperature)	LoD = 70 pg/mL [82] LoD = 1.55 pM [83] LoD = 0.26 pM [84] LoD = 0.13 pM [85] DR = 0.5 fM–100 pM LoD = 0.46 ± 0.006 fM [86]
Mechanical	Operates label-free Requires ~30 min for detection	Integrates well with PoC formats Easy to use	Highly sensitive to mechanical and thermal disturbances Complex fabrication processes	LoD = 7.6 pg/mL [87] LoD = 300 pM [88] LoD = 100 pM–DR = 6 ng/mL–60 µg/mL [89] LoD = 0.3 pM; Δf~50 Hz [90]
Optical/photonic	Requires little sample preparation Capable of real-time analysis Generally, it is more costly	Capable of real-time analysis Delivers high temporal resolution and S Provides consistent and reliable results. Suitable for low-cost integration High compatibility with miniaturized systems	Systems are typically bulky Performance may vary with environmental conditions	S = 4034 dB/RIU; LoD = 3.7 pg/mL [91] S~97, 51%, and 96.29% [92] LoD = 0.6 nM; Δλ~pm [93] LoD = 3 nM [94] LoD = 6.5 pM; Δλ = 15 pm [95]

**Figure 2:**
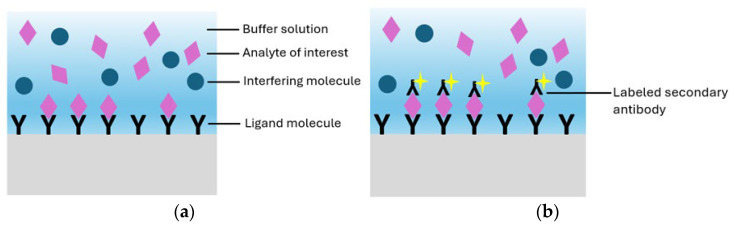
(**a**) Representation of a label-free biosensor and (**b**) a label-based biosensor.

Real-time read-out, label-free detection, low limits of detection, immunity to electromagnetic interference, and compatibility with minimally invasive sampling have driven their adoption in precision oncology assays [24,96]. Depending on the detection strategy, biosensor technologies can be categorized into systems that require external labels and those that operate without them. In label-free configurations, detection is achieved through direct biomolecular interactions at the sensor surface, typically inducing refractive index changes without the need for fluorescent or colorimetric tags (Figure 2a). This surface confined interaction, largely independent of sample volume, enables robust signal generation and real-time kinetic profiling. In contrast, label-based detection employs optical tags, such as fluorophores, quantum dots, or luminescent nanomaterials, to generate measurable signals through colorimetric, fluorescent, or luminescent mechanisms [97,98] (Figure 2b). Among these, green fluorescent protein (GFP), with an emission peak at 509 nm and excitation peaks at 395 and 475 nm, remains a standard tool due to its compatibility with intracellular environments and low phototoxicity. Although label-based sensors offer precise molecular labeling and enable subcellular resolution imaging, label-free photonic biosensors are increasingly favored for their operational simplicity, rapid analysis, and ability to track biomolecular events dynamically, eliminating the need for labeling and its associated limitations.

### 4.1. Photonic Biosensors: Principles and Technologies

**Figure 3:**
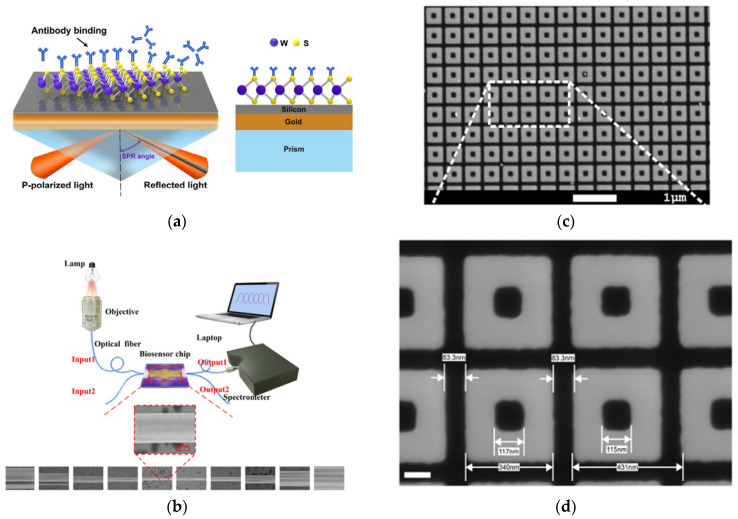
(**a**) Schematic representation of SPR biosensor. Adapted from [102] Licensed under CC BY 4.0; (**b**) experimental setup for optical fiber immunosensor-based system and a sequence of SEM images of the fabricated optical fiber. Adapted from [105] Licensed under CC BY-NC-ND 4.0; (**c**,**d**) SEM views of the nanocuboid array. Adapted from [110] Licensed under CC BY-NC-ND 4.0, [111].

Label-free photonic biosensors exploit the evanescent field generated by light propagation within confined optical structures, such as waveguides or nanostructured materials, allowing for the sensing of local variations in refractive index caused by biomolecular interactions. This optical detection mechanism supports direct and highly sensitive measurement of diverse analyte types, ranging from proteins and nucleic acids to small compounds and intact cells. [99,100]. Thanks to continuous advancements in nanophotonic and materials science, these technologies have rapidly evolved into a broad class of platforms tailored for biomedical applications. Four major categories of label-free photonic biosensors have emerged as particularly relevant for clinical diagnostics, such as LB: surface plasmon resonance (SPR), optical waveguide- and fiber-based systems, optical resonators, and metasurfaces. Each is characterized by distinct operating principles, sensitivity levels, and integration capabilities with microfluidic and chip-based systems. SPR platforms, the most mature among these technologies, offer high reliability, real-time monitoring, excellent sensitivity, and superior temporal resolution [101]. SPR is based on the excitation of surface plasmon waves, collective oscillations of free electrons, occurring at the boundary between a noble metal, typically gold, and a dielectric material, under well-defined optical conditions (Figure 3a) [102]. In recent years, the refinement of metallic interface design and the introduction of advanced surface coating have significantly improved the control over optical parameters such as refractive index and film thickness. These innovations have contributed to enhancing the performance of SPR-based sensors, making them more effective in detecting small-sized biomolecules (under 10 kDa) and in identifying target analytes present at extremely low concentrations (below 1 picomolar). Its high technological maturity and commercial readiness facilitate seamless adoption in clinical workflows [101,103]. The integration of nanostructures on sensing surfaces, such as nanoparticle arrays and nanohole arrays (NHAs), has enabled advanced detection mechanisms like localized surface plasmon resonance (LSPR) and extraordinary optical transmission (EOT), improving multiplexing, field confinement, and sensitivity.

Optical waveguide- and fiber-based biosensors exhibit low propagation losses and support a variety of geometries (slabs and channels), enabling strong optical confinement and efficient integration with microfluidic systems [104]. A representative setup includes a fiber-optic immunosensor with SEM imaging and laser-based biosensing (Figure 3b) [105]. A key challenge remains the trade-off between refractive index contrast and biocompatibility in polymer-based materials. Recent developments have increased the index contrast up to 10^−2^ RIU, significantly higher than the conventional 10^−3^ RIU, enhancing biosensor sensitivity [106] and making these platforms attractive for lab-on-chip (LoC) clinical applications. Optical resonator-based biosensors, such as whispering gallery mode (WGM) resonators, photonic crystals, and Fabry–Pérot interferometers (FPIs), offer ultra-high analytical sensitivity, real-time monitoring, and strong miniaturization and multiplexing capabilities [107,108]. A notable clinical example is the FDA-approved Maverick™ system, based on silicon microring resonators, used for simultaneous detection of SARS-CoV-2 IgG/IgM and ribonucleoproteins [109]. In recent years, metasurfaces have gained significant attention as versatile tools for biosensing, thanks to their nanometric control of light–matter interactions. They consist of subwavelength meta-atom arrays capable of manipulating electromagnetic wavefronts with high precision. For instance, a nanocube-based metasurface array (Figure 3c,d) was designed to trap and detect more than 1000 biological nanoparticles of 100 nm in diameter [110,111]. These structures offer precise control of phase, amplitude, and polarization in ultrathin formats [112]; dielectric metasurfaces provide sharp optical resonances, low losses, and CMOS compatibility, making them strong candidates for integrated refractometric sensors [113]. Despite their promising capabilities, clinical translation remains limited due to fabrication complexity. Current efforts are focusing on reconfigurable and programmable metasurfaces, capable of dynamic optical responses through structural tunability, paving the way for the development of next-generation compact, multifunctional biosensors [114].

Rather than favoring a single superior approach, photonic biosensing technologies exhibit functional complementarity, with each platform offering specific advantages based on the biomarker type, biological matrix, and clinical context. This diversity enables tailored biosensor designs suited to different operational needs. The field is thus evolving toward the integration of multiple optical modalities, combining their strengths to meet the demands of precision medicine.

### 4.2. Photonic Biosensors for Circulating Biomarkers

**Figure 4:**
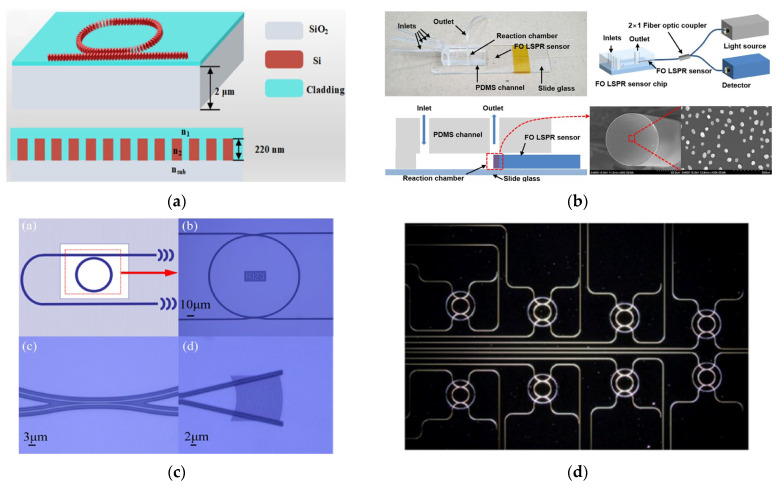
(**a**) Schematic representation and cross-section view of the sub-wavelength grating. Adapted from [115] Licensed under CC BY 4.0; (**b**) scheme and optical microscope images of MRR. Adapted from [118] Licensed under CC BY 4.0; (**c**) fabricated fiber-optic LSPR sensor with integrated view of the sensor chip, optical setup, and FEM images of the gold nanoparticle functionalize fiber tip. Adapted from [119] Licensed under CC BY 4.0; (**d**) dark-field optical microscopy image of MRR array. Adapted from [120] Copyright 2021 Elsevier.

Following the overview of key photonic technologies, this section highlights their application in detecting circulating biomarkers relevant to liquid biopsy through representative examples and performance metrics. For instance, L. Liu et al. [115] introduced a sub-wavelength grating racetrack microring resonator exploiting the Vernier effect, which enabled an S of 7061 nm/RIU and an LoD of 1.74 × 10^−5^ RIU (Figure 4a). Two distinct platforms have recently focused on the detection of immunoglobulin G (IgG), a clinically relevant biomarker in blood-based diagnostics. G. Brunetti et al. [116] proposed a dielectric metasurface composed of 2D dimer arrays exploiting the optical slot effect, achieving a Q-factor of 3.19 × 10^3^ and S of 25 nm/RIU for IgG detection in blood. In contrast, I. Del Villar et al. [117] developed an optical fiber biosensor based on Bloch surface wave resonances, generated by a non-dimensional photonic crystal on D-shaped single-mode fibers, reaching a detection limit of 70 attomolar for IgG in serum, demonstrating exceptional sensitivity for clinical applications. Additionally, X. Chen et al. [118] designed a microring resonator array for the label-free detection of human serum albumin (HAS), achieving an S of 168 nm/RIU and a LoD of 63.54 ng/mL, comparable to current state-of-the-art assays (Figure 4b). H. M. Kim et al. [119] reported a fiber-optic biosensor based on LSPR, functionalized with gold nanoparticles, for the detection of thyroglobulin (Tg). The sensor demonstrated high specificity and sensitivity, achieving a LoD of 93.11 fg/mL in patient serum samples (Figure 4c). Other clinically relevant indicators, sometimes associated with malignant neoplasms, include C-Reactive Protein (CRP) and Creatine Kinase-MB (CK-MB). In this context, R. Favaretto et al. [120] developed a MicroRing Resonator (MRR) platform for their simultaneous detection in filtered, undiluted artificial saliva, reporting promising sensitivity levels with LoDs of 10^3^ pM for CRP and 240 Pm for CK-MB (Figure 4d). Beyond traditional protein targets, they are increasingly applied to circulating biomarkers central to LC. Table 4 summarizes their analytical performance in liquid biopsy applications. Having established the core characteristics of photonic biosensing platforms for the main circulating biomarkers, the subsequent sections focus on clinically relevant tumor biomarkers: HER2, CEA, and PSA. As detailed in Section 2, these were selected due to the high incidence and clinical importance of prostate, breast, and lung cancers, which can be detected at early stages through these markers. For each biomarker, we provide a critical assessment of key photonic biosensors developed for their detection, highlighting analytical performance, technical feasibility, and clinical relevance.

**Table 4:** Summary of recent label-free photonic biosensor technologies applied to the detection of circulating biomarkers relevant to LB, including their performance metrics.

Photonic Biosensor Technologies	LB Biomarkers	Performance	Ref.
1D defective ternary photonic crystal	CTCs (glioblastoma)	Bulk sensitivity = 4170.34 nm/RIU	[121]
SERS sensors	CTCs	LoD < 1 cell mL^−1^	[122]
SPR sensors	miRNA	LoD = 1 × 10^−18^ M	[123]
SPR sensors	Exosomes	LoD ranging from 5000 to 10,000 exosomes per µL	[124,125]
LSPR	Exosome	Single exosome detection	[126]
WGM/PhC optical resonators	cfRNA	LoD = 10 × 10^−12^ M	[127]
LSPR	cfRNA	LoD~10 × 10^−15^ M	[128]

### 4.3. Photonic Biosensors for Detection of HER2

**Figure 5:**
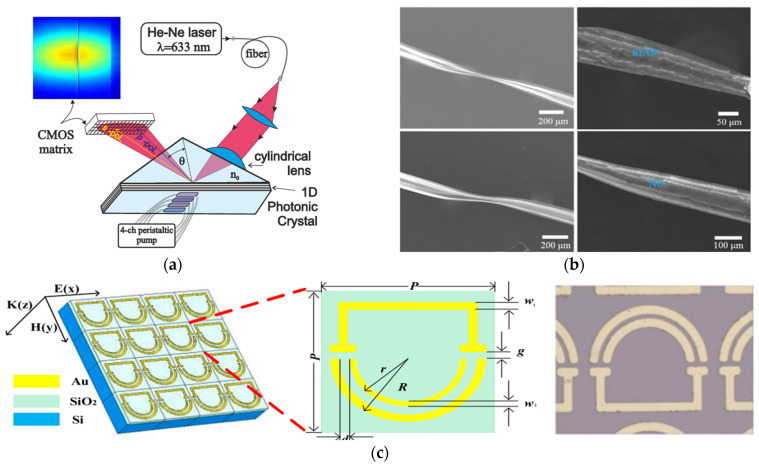
(**a**) Schematic diagram of the EVA 2.0 sensor system. Adapted from [131] Licensed under CC BY 4.0; (**b**) SEM image sequence illustrating the evolution of the surface thin film (STF) morphology in mAb- and Nb-based immunosensors. Shown are the STF structures before and after antibody or nanobody functionalization in both sensor types. Adapted from [133] Copyright 2024 Elsevier; (**c**) local and top views of THz metasurface biosensor. Adapted from [135] Copyright 2022 Elsevier.

HER2 is a transmembrane tyrosine kinase overexpressed in approximately 15% of breast cancer cases and is linked to more aggressive disease progression and less favorable clinical outcomes [129]. Although it is not currently recommended to guide adjuvant therapy decisions, circulating HER2 is a promising marker for disease surveillance, relapse detection, and response assessment in advanced BC. This clinical relevance has driven the design of advanced photonic biosensing technologies tailored to the detection of HER2 in body fluid within the framework of LB [130]. In this context, I. Petrova et al. [131] developed EVA 2.0, a planar 1D photonic crystal biosensor exploiting Photonic Crystal Surface Modes (PCSMs) for label-free and multiplexed detection of HER2 (Figure 5a). Fabricated on a BK-7 glass substrate with alternating layers of SiO_2_ (233.1 nm) and Ta_2_O_5_ (72.5 nm) deposited via magnetron sputtering, the sensor demonstrated exceptional analytical performance with a LoD of 630 fg/mL and a linear range extending up to 50 pg/mL for HER2, substantially outperforming conventional gold nanoparticle-enhanced SPR platforms. PCSM architecture enables high sensitivity and multiplexing capability on a single chip, positioning EVA 2.0 as a highly promising tool for real-time, multiplexing analysis in clinical diagnostics.

While EVA 2.0 represents a significant advance in PCSM-based photonic biosensing, recent SPR innovations are also noteworthy. Notably, L. Rahmidar et al. [132] developed a label-free SPR biosensor functionalized with zirconium-based metal–organic frameworks, specifically UiO-66 and UiO-66-NH_2_, to enhance binding affinity and sensitivity for HER2 detection. The incorporation of metal–organic frameworks (MOFs) onto the SPR chip not only improved surface functionalization but also enabled an LoD of 0.457 ng/mL with UiO-66-NH_2_, well below clinically relevant HER2 concentrations. Although not achieving the ultralow detection limits of EVA 2.0, the MOF-functionalized SPR biosensor demonstrates a compelling balance between sensitivity, chemical stability, and ease of integration, supporting its use in early diagnostic strategies for breast cancer, especially in settings where multiplexing is not required. Beyond SPR and PCSM-based platforms, optical fiber and metasurface technologies have also shown considerable promise for HER2 detection, leveraging innovative material integration and advanced sensing configurations. For instance, W. Guo et al. [133] developed a novel immunosensor utilizing a modified S-tapered optical fiber (STF) coated with gold nanoparticles and graphene oxide for the label-free quantification of HER2 (Figure 5b). A major advancement involves the simultaneous utilization of monoclonal antibodies (mAbs) and nanobodies (Nbs) to functionalize the sensing surface, enabling a comparative performance analysis. The Nb-based configuration achieved an exceptional LoD of 0.001 nM, markedly surpassing the sensitivity of its mAb counterpart. This result highlights the potential of nanobody functionalized optical fibers in offering both high sensitivity and physical miniaturization and flexibility, attributes particularly advantageous for PoC diagnostics. Furthermore, photonic crystal-based biosensors have demonstrated outstanding performance in the detection of HER2. A. Molaei-Yeznabad et al. [134] proposed a photonic crystal biosensor that detects HER2 and CEA via refractive index modulation, leading to spectral shifts in transmission. The sensor demonstrated an S of 692.8 nm/RIU, FOM of 2561.5 RIU^−1^, and a Q-factor exceeding 5870, indicating remarkable spectral resolution and signal-to-noise performance. Compared to fiber-optic approaches, this design offers greater integration potential with chip-scale platforms and is cost effective, although its specificity and functionalization versatility may be more limited than that of Nb-functionalized sensors. In parallel, terahertz (THz) metasurfaces have emerged as an alternative photonic platform, combining structured nanophotonics with high surface functionalization capabilities. Q. Zeng et al. [135] developed an aptamer-modified THz metasurface biosensor composed of split-ring resonators (SRRs) (Figure 5c), with a 500 nm SiO_2_ spacer layer inserted to enhance the Q-factor. This design achieved a LoD of 0.1 ng/mL for HER2, outperforming conventional SRRs fabricated directly on silicon. Its ability to detect additional analytes such as CEA and Alpha-fetoprotein (AFP) further underscores the multiplexing potential and versatility of this class of biosensors. In general, graphene-based metasurfaces have demonstrated high sensitivity in detecting circulating biomolecules, which have proven particularly effective for early detection and prognosis of cancer [136]. Building on recent advances in metasurface engineering, S.K. Patel et al. [137] introduced a platform based on a gold nano-octagonal metastructure integrated with graphene, aimed at enhancing optical absorption and biomolecular sensitivity.

This biosensor could identify circulating cancer-related markers in human serum with high sensitivity to small concentration differences, reaching a maximum S of 800 GHz/RIU and an LoD of 0.328 RIU^−1^. While this platform may not match the absolute LoD achieved by some nanobody- or aptamer-based sensors, its ability to detect even the smallest change in the refractive index makes it perfect for uses where early deviations from standard values are important for diagnosis. Diverse photonic strategies for HER2 detection leverage distinct physical principles, highlighting complementary strengths. Optimal sensor design requires careful trade-offs among sensitivity, specificity, multiplexing capacity, and system integrability rather than a universal design. This convergence enhances both technological innovation and clinical applicability in precision oncology.

### 4.4. Photonic Biosensors for Detection of CEA

**Figure 6:**
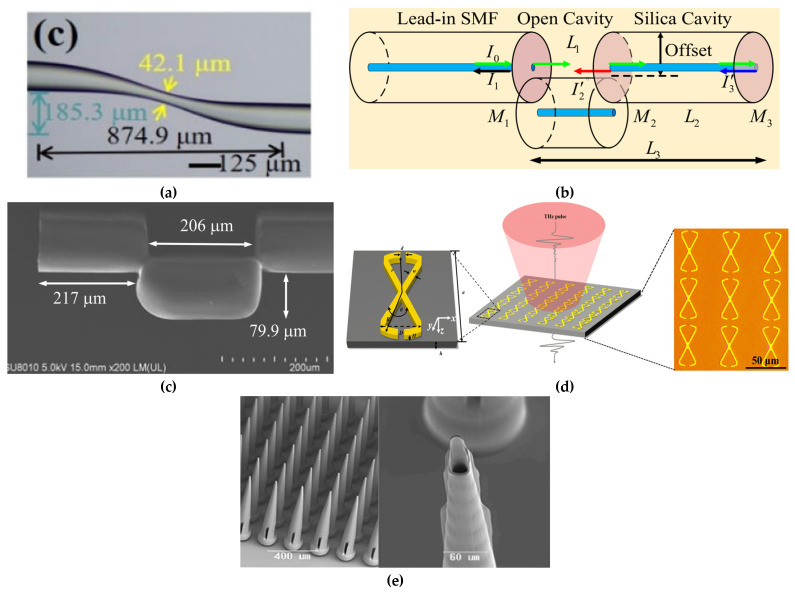
(**a**) Optical microscope image of fiber immunosensor. Adapted from [143] Copyright 2023 IEEE; (**b**) schematic representation of the open-cavity FPI. Adapted from [144] Copyright 2024 Elsevier; (**c**) SEM images illustrating the structural features of the fabricated senso.Adapted from [144] Copyright 2023 IEEE; (**d**) schematic representation of the toroidal metasurface and a microscope image of the fabricated toroidal unit cells. Adapted with permission from [145]. Copyright 2022 American Chemical Society; (**e**) SEM images of microneedle array. Adapted from [146] Licensed under CC BY 4.0.

CEA is a glycoprotein widely recognized as a tumor-associated biomarker, with elevated serum levels observed in various malignancies, including lung cancer [138]. Due to its clinical relevance in disease prognosis and therapeutic monitoring, CEA has attracted substantial interest as a target for advanced label-free and high-sensitivity photonic biosensing platforms. Among the most prominent technologies, LSPR- and SPR-based biosensors have been extensively explored also for CEA quantification. For instance, Z. Ma et al. [139] developed a microfluidic integrated LSPR biosensor incorporating multiple sensing units and gold nanoparticles, achieving a detection range of 0.5–100 ng/mL and a LoD below 1 ng/mL. System optimization enabled an 85-fold improvement in the signal-to-noise ratio (SNR), delivering ELISA-comparable performance and underscoring LSPR’s early diagnostic potential. Building on this, R. Li et al. [140] introduced a streptavidin-modified SPR biosensor employing gold nanoparticles in a sandwich configuration. This enhancement significantly improved specificity and sensitivity, achieving a LoD of 1.0 ng/mL and S gain of 13.8 times compared to nanoparticle-free formats. Importantly, it maintained high selectivity even in the presence of interfering biomarkers such as AFP and PSA. Further advancements in materials engineering and nanophotonics have enabled alternative architectures with enhanced performance. A. Kumar et al. [141] proposed a graphene-enhanced SPR biosensor incorporating MXene and MoS_2_ layers between silver and graphene films, resulting in improved electromagnetic field confinement and a sensitivity of 144.72 deg./RIU. This multilayer design illustrates how 2D materials can be strategically employed to augment SPR platforms beyond conventional configurations. In terms of leveraging waveguide optics, H. Mukundan et al. [142] developed a SiON_X_ planar waveguide biosensor capable of detecting sub-picomolar levels of CEA (<0.5 pM) in serum and NAF. This device achieved both high sensitivity and minimal sample consumption (10 µL), making it well suited for multiplex and PoC applications. The biofunctionalization process involved avidin–biotin chemistry and SiO_2_ coating to ensure surface stability and antibody immobilization, showcasing how photonic integration and surface chemistry can be jointly optimized. Fiber-optic biosensors have also demonstrated impressive results. Y. Wei et al. [105] designed an optical microfiber coupler (OMC)-based immunosensor, integrated into a microfluidic chip, achieving a remarkably low LoD of 34.6 fg/mL (0.475 fM). The use of a halogen lamp, microscope optics, and a 0.3 nm resolution spectrometer allowed precise spectral measurements and reliable quantification across clinically relevant ranges. Similarly, W. Guo et al. [143] explored optical fiber microstructures functionalized with graphene oxide (GO) and gold nanoparticles (GNPs) through a layer-by-layer assembly process (Figure 6a), achieving sensitivities of 15.1 nm/nM (GO) and 20.2 nm/nM (GNPs) and LoDs of 0.05 nM and 0.02 nM, respectively. The use of nanobodies instead of conventional antibodies contributed to faster response times and improved selectivity, demonstrating the value of miniaturized and nanostructured optical fiber platforms for rapid CEA detection. Innovations in interferometric and metamaterial-based sensing further broaden the design space. L. Chen et al. [144] reported an open-cavity FPI enhanced by the Vernier effect, functionalized with anti-CEA antibodies (Figure 6b,c). This configuration achieved a LoD of 36.14 fg/mL and demonstrated high stability, reproducibility, and specificity, with response times under 30 min. Complementarily, Q. Niu et al. [145] introduced a THz metamaterial biosensor integrating gold nanoparticles to exploit the unique properties of toroidal electromagnetic modes (Figure 6d).The device exhibited an S of 287.8 GHz/RIU, a Q-factor of 15.04, and a LoD of 0.17 ng, offering a powerful platform for CEA analysis. In contrast to the THz toroidal biosensor by Q. Niu et al. [145], S. Geng et al. [146] developed a photonic metamaterial platform integrating near-field enhancement and wavelength selectivity for minimally invasive detection of CEA, CA125, and CYFRA21-1 in interstitial fluid (Figure 6e). This system outperforms conventional sensors in sensitivity and resolution, offering a faster, scalable alternative for early cancer diagnostics despite fabrication complexities. The development of photonic biosensors for CEA detection reveals not only technological innovation but also strategic differentiation, with platforms optimized for distinct clinical and operational contexts.

### 4.5. Photonic Biosensors for Detection of PSA

PSA remains the most widely used molecular indicator for prostate cancer (PCa) screening; however, its limited specificity often leads to false positives, unnecessary biopsies, and overdiagnosis [147]. To address these limitations, various photonic biosensing platforms have been proposed to enhance the analytical performance of PSA detection in the context of LB. Among label-free plasmonic approaches, M. Mahani et al. [148] demonstrated a GNP-based biosensor capable of detecting ultra-low PSA concentrations in serum, leveraging a colorimetric shift induced by nanoparticle aggregation. The sensor, with an S of 43.75 nm/ng·mL^−1^, exploits LSPR redshift phenomena caused by refractive index changes upon antigen–antibody binding. While effective, this approach may face limitations in multiplexing and real-time integration. A more refined SPR-based strategy was proposed by R. Li et al. [140], who enhanced specificity through streptavidin-modified GNPs (SA-GNPs) in a sandwich biosensor configuration. Although their application focused on CEA, the system achieved a 13.8-fold improvement over traditional formats and showed no cross-reactivity with PSA, suggesting potential adaptability for PSA assays. Resonance-enhanced photonic sensors have likewise emerged as competitive alternatives. X. Dai et al. [149] developed a dual-resonance lossy mode resonance (LMR) immunoprobe, integrating MgF_2_ and ITO layers to optimize phase matching and resonance quality.

**Figure 7:**
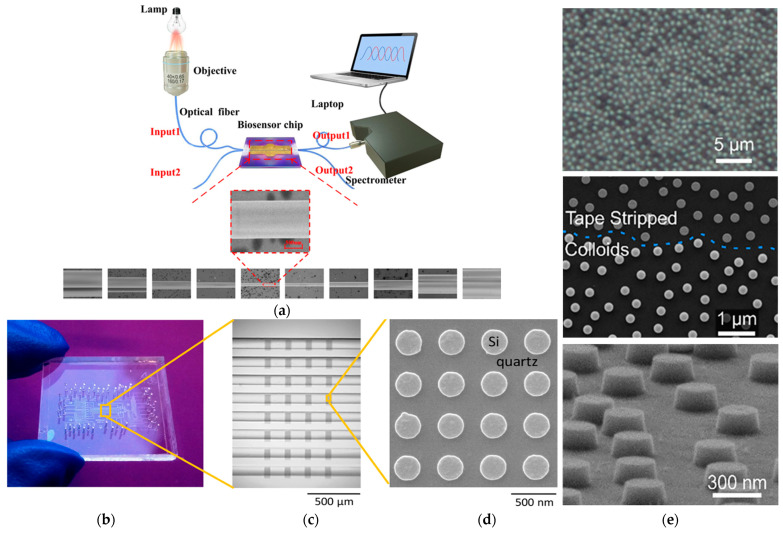
(**a**) Schematic diagram of fiber laser-based biosensor system. Adapted from [105] Licensed under CC BY-NC-ND 4.0; (**b**) biosensing system based on refractometric method that presents eight flow channels integrated with silicon nanodisk array. Adapted with permission from [153]. Copyright 2017 American Chemical Society; (**c**) SEM microscope image of nanocylinders before and after substrate cleaning with piranha solution. Adapted with permission from [153]. Copyright 2017 American Chemical Society; (**d**) SEM images of the silicon nanodisk. Copyright 2017 American Chemical Society; (**e**) SEM images of the arrays of silicon nanocylinders. Adapted with permission from [154]. Copyright 2019 American Chemical Society.

The sensor’s sensitivity is 206.657 nm/RIU and has a LoD of 52 pg/mL, coupled with a 90% diagnostic recognition rate in clinical samples, demonstrating the value of dual resonance schemes for high-precision, portable PCa screening. In the realm of fiber-optic systems, A.Z.M. Zamri et al. [150] introduced a D-shaped, gold-coated fiber laser biosensor, achieving an S of 0.2171 nm/(µg/mL) for PSA. Its integration with a 980 nm laser diode and WDM unit, monitored via an Optical Spectrum Analyzer (OSA), illustrates the potential for compact, real-time diagnostics (Figure 7a). Nanophotonic platforms further broaden the sensing landscape. M. Shokorlou et al. [151] proposed a metal–insulator–metal (MIM) nanoring sensor with an S of 567.23 nm/RIU and an FOM of 3.72, offering a miniaturized, responsive format compatible with integrated systems. Similarly, T. Zhang et al. [152] proposed an arrayed nanostructured FPI microchip for the label-free detection of free PSA (f-PSA), achieving a LoD of 10 pg/mL. The dynamic range of the sensor was tunable through precise antibody immobilization, enabling tailored analytical response for different clinical scenarios. Collectively, these technologies underscore the diversity of optical strategies for PSA detection, from plasmonic and interferometric platforms to waveguide-based and nanostructured sensors. Each architecture provides complementary trade-offs among sensitivity, specificity, integration complexity, real-time operation, and multiplexing capability. In addition to conventional plasmonic and interferometric configurations, dielectric metasurfaces have recently attracted growing interest for PSA analysis owing to their excellent spectral resolution and suitability for integration into LoC systems. Notably, O. Yavas et al. [153] proposed two silicon metasurface platforms combining nanophotonic sensitivity with microfluidic compatibility. The first design leveraged Electron Beam Lithography (EBL) to fabricate Si nanoantenna arrays operating in the near-infrared region, integrated within an LoC system featuring eight independent sensing channels (Figure 7b–d). This platform achieved a bulk S of 227 nm/RIU and an FOM of 5.4 RIU^−1^, with an LoD of 0.69 ng/mL, well within clinical thresholds for PSA quantification in serum. Although the system offered high performance, its reliance on EBL may limit scalability. To address this, the authors also developed a cost-effective metasurface fabricated via colloidal lithography, comprising Si nanocylinders exhibiting Mie resonances at 900 nm in aqueous environments (Figure 7e). This alternative showed an S of 86 nm/RIU and used both redshift and extinction reduction as transduction mechanisms [154]. Despite a slightly lower Q-factor, this architecture demonstrated competitive LoD values and suitability for scalable production. Together, these metasurface-based biosensors underscore the growing feasibility of integrating nanophotonic precision with practical manufacturability in PSA detection strategies for clinical use. Overall, these innovations demonstrate that photonic biosensors offer a strategic response to the long-standing trade-off between sensitivity and specificity in PSA testing. Their continued advancement is essential not only for improving diagnostic precision but also for addressing the clinical challenges of overdiagnosis and unnecessary interventions in prostate cancer screening.

## 5. Comparative Analysis of Photonic Biosensing Platforms

Table 5 presents a comparative overview of the photonic biosensing technologies reviewed, detailing their key characteristics and analytical performance indicators. This comparison helps evaluate the diagnostic value and technological suitability of each sensing technology for clinical applications.

No single biosensing technology outperforms all others across every performance parameter. Each technology offers unique advantages and limitations that must be weighed according to the specific clinical context and target biomarker. Optical fiber- and waveguide-based biosensors are particularly notable for their ultra-low LoDs, rapid response times, and high analytical precision. These characteristics make them ideal for PoC diagnostics and early-stage cancer detection.

Optical resonators, especially those based on WGM and photonic crystal architectures, demonstrate the highest levels of sensitivity and Q-factors, highlighting their strong potential for high-precision analyses. These platforms also offer robust and cost-effective sensing solutions, particularly when integrated within scalable photonic systems. SPR and LSPR sensors remain among the most widely adopted technologies. Their widespread adoption is attributable to their maturity, real-time and label-free detection capabilities, multiplexing potential, and compatibility with nanometric enhancements. Meanwhile, metasurface-based sensors, though still emerging, show promising performance and versatility and offer significant potential for miniaturization and integration. Overall, Table 5 highlights the importance of selecting the most suitable photonic sensing technology not only based on analytical performance but also on clinical objectives and biomarker characteristics.

## 6. From Technological Innovation to Clinical Applicability: Current Challenges and Future Prospective

The integration of innovative biosensing technologies has played a pivotal role in accelerating the clinical implementation of PoC applications, such as LB. Indeed, the global marker for PoC diagnostics, driven by continuous advancements in sensor platforms, was valued at USD 46.65 billion in 2021, with projections indicating a rise to USD 51.94 billion by 2029, corresponding to a compound annual growth rate (CAGR) of 5.2%. Despite this progress, integrating LB into routine clinical practice remains a complex challenge, hindered by several technological, organizational, and regulatory barriers. From a technical perspective, achieving sufficient sensitivity and specificity is particularly challenging [61]. The lack of standardized protocols for isolating exosomes and other vesicle- or nucleic acid-based biomarkers compromises the reproducibility and reliability of results. Additionally, the inherent instability of certain biomarkers, notably ctDNA, with a half-life of approximately 1.5 to 2 h in peripheral blood [155], makes strict pre-analytical handling essential. However, such protocols are not yet fully developed or widely implemented [27].

Equally important is the need for appropriate training of clinical and laboratory personnel, including physicians, pathologists, nurses, and technicians, not only in the operation of diagnostic platforms but also in interpreting their results accurately. This is especially relevant in settings where testing is outsourced to centralized laboratories. Accordingly, training should be incorporated into both academic curricula and professional development pathways and supported by structured continuing education programs [156]. Standardizing procedures from sample collection to storage is also critical to ensuring consistency across institutions. However, in some clinical settings, there may be resistance to adopting new technologies. This unwillingness often stems from long-standing trust in traditional methods, which are well established, widely validated, and familiar to practitioners. Furthermore, without sufficient clinical infrastructure and support, even the most advanced technologies risk remaining peripheral to routine medical practice. Finally, clinical validation and regulatory approval remain significant bottlenecks to the adoption of LB and biosensors technologies. Many biosensor-based LB tests have yet to undergo the large-scale clinical validation needed to demonstrate their efficacy and clinical utility. At the same time, the absence of a clear regulatory framework delay approval and reimbursement, limiting both access and economic sustainability. In this context, initiatives such as the European CANCER-ID [157] and the European Liquid Biopsy Society (ELBS) [158] are actively working to address these gaps by promoting the harmonization of procedures and the establishment of quality standards essential for full clinical translation.

Identifying the main challenges currently limiting the clinical applicability of LB is essential to define strategic research priorities. The use of AI is posed to optimize LB assay performance and streamline their adoption in healthcare systems [159]. Notable examples include the use of machine learning for detecting and characterizing CTCs, analyzing ctDNA for cancer diagnosis and localization, and integrating LB results with metabolomic, immunologic, microbiomics, and homeostatic data to support clinical decision making [160]. A notable example is Lung-CLiP, a machine learning algorithm developed to analyze plasma cfDNA alongside matched leukocyte DNA. Trained on well-annotated clinical and imaging datasets, including PET-CT-based metabolic tumor volume (MTV), the model has shown promising results in distinguishing early-stage NSCLC from high-risk controls. It achieved sensitivity levels ranging from 16% to 80%, depending on tumor volume, with a constant specificity of 98% [161]. Concurrently, the advancement of biosensor technologies must be guided by several key research directions. Enhancing analytical parameters, such as sensitivity, reproducibility, and detection limits, remains a top priority [162]. Additionally, future research should emphasize the miniaturization of biosensors, which is crucial for enabling minimally invasive, cost-effective, and portable diagnostic tools [163]. Their integration into advanced platforms like LoC systems will further support the development of rapid, decentralized PoC diagnostics [18]. Ensuring biosensor functionality in complex biological environments is equally important, as these conditions can compromise stability and accuracy. To guarantee clinical reliability, the standardization of preanalytical variables, including sampling, storage, and processing, is critical. Lastly, fostering data sharing and academic-industrial collaboration will accelerate innovation and bring LB technologies into routine clinical practice [164]. Beyond the refinement of core photonic sensing architectures, future research should also consider the integration of complementary technologies that may enhance the analytical capabilities and clinical usability of biosensors. Notably, the Clustered Regularly Interspaced Short Palindromic Repeats (CRISPR-Cas9) system has emerged as a powerful molecular recognition tool [165]. Thanks to its high specificity in targeting nucleic acid, it can be incorporated into CRISPR-based optical biosensors as a programmable element for detecting genetic biomarkers. When coupled with photonic transduction mechanisms, such systems offer enhanced sensitivity and target selectivity, particularly in applications involving ctDNA and RNA markers [166]. Lastly, isothermal amplification techniques, such as LAMP or RPA, provide efficient alternatives to PCR [167]. These methods enable amplification at constant temperatures, eliminating the need for complex thermal cycling equipment. As a result, they are well suited for integration with photonic biosensors to improve detection thresholds and facilitate miniaturization and field deployment.

With ongoing advances in research and technology, LB is expected to become an integral part of routine clinical screening. Nevertheless, its clinical uptake has been relatively slow and still requires significant refinement. As with any emerging innovation, the successful transition from cutting-edge sensing platforms to widespread clinical application demands not only solid technological validation but also a cultural and educational shift within the medical community.

## 7. Conclusions

Over the past decades, the biomedical research community has increasingly focused on developing novel, non-invasive diagnostic strategies to improve the accuracy, efficiency, and patient compliance of cancer diagnosis and prognosis. Within this paradigm, LB has gained prominence as a game-changing technique, allowing for the identification of circulating tumor cells and other cancer-associated biomarkers in biological fluids. This strategy provides a minimally invasive and potentially more effective alternative to conventional tissue biopsy [9,10]. Photonic biosensors, including platforms based on plasmonic structures [103] and SOI technologies, are reshaping the analytical landscape of LB by providing highly sensitive, specific, and rapid detection platforms tailored to clinically relevant biomarkers. By evaluating sensing technologies in relation to biomarker-specific diagnostic requirements, this review highlights the importance of aligning sensor design with both biological targets and clinical utility. The strategic deployment of photonic architectures adapted to distinct diagnostic scenarios represents a promising route toward clinical translation [156,164]. Achieving this goal will require sustained interdisciplinary collaboration across photonics, biology, oncology, and bioengineering, which will be essential to transition these technologies from experimental prototypes to standardized tools in precision oncology.

## Figures and Tables

**Table 1:** Comparison of key advantages and disadvantages of LB and conventional tissue biopsy.

	Advantages	Disadvantages
Liquid biopsy	Minimally intrusive and readily accessible procedure [18] Can be used for regular and repeated monitoring during treatment and disease progression, allowing for dynamic follow-up of tumor evolution [18] Enables genomic analysis of ctDNA or CTCs, facilitating the repeated evaluation of clonal dynamics during treatment and early detection of factors driving therapeutic resistance [13,14] Faster processing time compared to tissue biopsy [18]	Risk of false negative results [19] Variations in cfDNA levels among patients may compromise the accuracy and reliability of the tests [13] Extensive validation required: ultrasensitive detection and identification of mutations necessitate extensive validation to ensure that the results are clinically actionable and reliable [12] Need for highly sensitive techniques [13]
Conventional tissue biopsy	Allows detailed histological analysis and accurate disease staging, providing critical information for diagnosis and treatment planning [13] Conventional tissue biopsy remains essential for characterizing tumors, enabling classification by cancer type, the evaluation of tumor-specific gene expression, and the detection of mutations associated with therapy resistance [18] Gold standard due to its high success rate in providing comprehensive and reliable diagnostic information [18] It is an accurate method for its thoroughness and accuracy in disease assessment [18]	Invasive approach: source of discomfort for patients with several risks of clinical complications [13] Limited to localized tissue analysis, does not enable assessment of intra-or inter-tumor heterogeneity (metastases) [11]. Local anesthetic is required [13] Image-guided surgery system is required [6] No real-time monitoring [11] No possibility of dynamic follow-up of cancer molecular modifications [13]

**Table 5:** Comparative summary of photonic biosensing technologies for cancer biomarker (HER2, CEA, and PSA) detection.

Biomarker	Technology	Biological Sample	Performance	Notes	Ref.
HER	SPR	Blood serum	LoD = 630 fg/mL	Multiplexing, real-time detection	[131]
	SPR	Blood serum	S = ng/mL LoD = 0.457 ng/mL	Multiplexing	[132]
	Optical fiber/WG	Blood serum	LoD = 0.001 nM	Extremely high S	[133]
	Optical resonator (photonic crystal)	Suspension of normal and cancerous cells	S = 692.8 nm/RIU	Q-factor = 5870 FOM = 2561.5 RIU^−1^ Robust and cost-effective platform	[134]
	Metasurface	HER standard solution	LoD = 0.1 ng/mL	Higher S compared to conventional SRRs, high versatility	[135]
CEA	LSPR	Human serum	S = 85 × SNR LoD < 1 ng/mL	Integrated with microfluidics, with the use of GNPs	[139]
	SPR	Human serum	S = 13.8× with respect to std configuration LoD = 1.0 ng/mL	Good selectivity (no interference from other tumor biomarkers)	[140]
	SPR	CEA standard solution (PBS solution)	S = 144.72 deg./RIU	Integrates MXenes and MoS2 between graphene and silver layers	[141]
	Optical fiber/WG	Human serum/NAF	LoD < 0.5 pM	High S, reaction time < 15 min, requiring 20 µL of NAF	[142]
	Optical fiber/WG	Human serum	LoD = 34.6 fg/mL (0.475 fM)	High S and wide dynamic range	[105]
	Optical fiber/WG	Human serum	S = 20.2 nm/nM (GNPs) S = 15.1 nm/nM (GO) LoD = 0.02 nM (GNPs) LoD = 0.05 nM (GO)	High specificity and rapid response	[143]
	Optical resonator (photonic crystal)	Suspension of normal and cancerous cells	S = 692.8 nm/RIU	Q-factor = 5870 FOM = 2561.5 RIU^−1^ Robust and cost-effective platform	[134]
	Optical resonator (Fabry-Perot)	No real fluid (purified CEA)	LoD = 36.14 fg/mL	Excellent stability, reproducibility, specificity, S, and time response < 30 min	[144]
	Metasurface	No real fluid (purified CEA)	S = 287.8 GHz/RIU LoD = 0.17 ng	Q-factor = 15.04 Excellent specificity	[145]
PSA	LSPR	Human serum	S = 43.75 nm/ng⋅mL^−1^ LoD = ng/mL	With the use of GNPs	[148]
	Optical fiber/WG	Human serum	S = 206.657 nm/RIU LoD = 52 pg/mL	High precision, 90% diagnostic rate	[149]
	Optical fiber/WG	Human serum	S = 0.2171 nm/(µg/mL)	Good sensibility	[150]
	Optical resonator (WGM)	Standard solutions in water	S = 567.23 nm/RIU	FOM = 3.72; compact platform	[151]
	Metasurface	Human serum	S = 227 nm/RIU LoD = 0.69 ng/mL	High integrability FOM = 5.4 RIU^−1^	[153]
	Metasurface	Human serum	S = 86 nm/RIU	Excellent S and a favorable LoD	[154]

## Data Availability

Data are available on request from the authors.

## Abbreviations

The following abbreviations are used in this manuscript:

LB	Liquid Biopsy
WHO	World Health Organization
CT	Computed Tomography
MRI	Magnetic Resonance Imaging
cfDNA	Circulating Cell-Free DNA
cfRNA	Circulating Cell-Free RNA
CTCs	Circulating Tumor Cells
cfmiRNAs	Cell-Free MicroRNAs
EVs	Extracellular Vesicles
TEPs	Tumor Educated Platelets
PoC	Point of Care
FP	Fabry–Perot
SOI	Silicon on Insulator
PCM	Phase-Changing Material
VO_2_	Vanadium Dioxide
PET	Positron Emission Tomography
FDA	Food and Drug Administration
MVs	Microvesicles
dPCR	Digital Polymerase Chain Reaction
BEAMing	Bead Emulsion Amplification Magnetics
TAmSeq	Tagged Amplicon Deep Sequencing
NGS	Next Generation Sequencing
miRNA	microRNA
mRNA	Messenger RNA
LOC	Lab-on-Chip
µTASs	Micro Total Analysis Systems
ddPCR	Droplet Digital PCR
DBS	Dried Blood Spot
VAMS	Volumetric Absorptive Microsampling
NAATs	Nucleic Acid Amplification Tests
CSF	Cerebrospinal Fluid
qRT-PCR	Real-Time Polymerase Chain Reaction
IVD	In Vitro Diagnostics
ACS	American Cancer Society
CDC	Centers for Disease Control and Prevention
NCI	National Cancer Institute
NAACCR	North American Association of Central Cancer Registries
PSA	Prostate Specific Antigen
CEA	Carcinoembryonic Antigen
HER 2	Epidermal Growth Factor Receptor 2
BC	Breast Cancer
IHC	Immunohistochemical
ER	Estrogen Receptor
PR	Progesterone Receptor
ELISA	Enzyme-Linked ImmunoSorbent Assay
MCF-7	Michigan Cancer Foundation-7
PAI-1	Plasminogen Activtor- 1
NAF	Nipple Aspirate Fluid
PC	Prostate Cancer
DRE	Digital Rectal Examination
NSCLS	Non-Small-Cell Lung Cancer
SCLC	Small-Cell Lung Cancer
BASs	Bronchial Aspirates
BAL	Bronchial Lavage
DR	Dynamic Range
KRAS	Kirsten Rat Sarcoma
PCR	Polymerase Chain Reaction
HPLC	High-Performance Liquid Chromatography
GFP	Green Fluorescent Protein
IgG	Immunoglobulin G
LoD	Limit of Detection
HAS	Human Serum Albumin
LSPR	Localized Surface Plasmon Resonance
Tg	Thyroglobulin
CRP	C-Reactive Protein
CK-MB	Creatine Kinase-MB
MRR	Microring Resonator
SPR	Surface Plasmon Resonance
NHAs	Nanohole Arrays
EOT	Extraordinary Optical Transmission
WGM	Whispering Gallery Mode
FPIs	Fabry–Perot Interferometers
PCSMs	Photonic Crystal Surface Modes
MOFs	Metal–Organic Frameworks
STF	S-Tapered Optical Fiber
mAb	Monoclonal Antibody
Nb	Nanobody
SRRs	Split-Ring Resonators
AFP	Alpha-Fetoprotein
STF	Surface Thin Film
SEM	Scanning Electron Microscope
SNR	Signal-to-Noise Ratio
OMC	Optical Microfiber Coupler
GO	Graphene Oxide
GNP	Gold Nanoparticle
SA-GNPs	Streptavidin-Modified Gold Nanoparticles
LMR	Lossy Mode Resonance
WDM	Wavelength Division Multiplexing
OSA	Optical Spectrum Analyzer
MIM	Metal–Insulator–Metal
FOM	Figure of Merit
f-PSA	Free Prostate-Specific Antigen
EBL	Electron Beam Lithography
CAGR	Compound Annual Growth Rate
ELBS	European Liquid Biopsy Society
AI	Artificial Intelligence
MTV	Metabolic Tumor Volume
NSCLC	Non-Small-Cell Lung Cancer
CRISPR-Cas9	Clustered Regularly Interspaced Short Palindromic Repeats
S	Sensitivity
